# Stimuli-Responsive Star Polymer as an Admixture for Crystallization of Hollow Crystals

**DOI:** 10.3390/ma15228240

**Published:** 2022-11-20

**Authors:** Marcin Lemanowicz, Justyna Chrzanowska, Milena Kotek, Anna Mielańczyk, Maria Kupczak, Daria Niewolik, Anna Korytkowska-Wałach, Olesya Klymenko, Alicja Kocur, Dorota Neugebauer

**Affiliations:** 1 Department of Chemical Engineering and Process Design, Faculty of Chemistry, Silesian University of Technology, ks. M. Strzody 7, 44-100 Gliwice, Poland; 2 Department of Physical Chemistry and Technology of Polymers, Faculty of Chemistry, Silesian University of Technology, ks. M. Strzody 9, 44-100 Gliwice, Poland; 3 Department of Organic Chemistry, Bioorganic Chemistry and Biotechnology, Faculty of Chemistry, Silesian University of Technology, B. Krzywoustego 4, 44-100 Gliwice, Poland; 4 Department of Histology and Embryology, School of Medicine with the Division of Dentistry in Zabrze, Medical University of Silesia, ul. Jordana 19, 41-808 Zabrze, Poland

**Keywords:** stimuli-responsive polymer, star polymer, crystallization, hollow crystals, KCl

## Abstract

Polymers are becoming a very popular tool in the crystallization of different compounds. In this work, a new method of crystallization is proposed using stimuli-responsive star polymer in order to obtain hollow structure crystals. In these experiments, amphiphilic copolymer of acrylic acid (AA) and methyl acrylate (MA) were used for isohydric crystallization via they cooling of KCl in deionized water solution. The experiments were realized in quartz cuvette with a magnetic stirrer using a specialized spectrometer with precise temperature control. The crystallization course was monitored by the absorbance readings and analysis of the nucleation energetic effect. It was proved that the moment of the polymer’s phase transition occurrence had an important role in the crystal growth process. On the other hand, the occurrence of phase transition did not trigger the nucleation. The supercoolings achieved in the presence of the polymer were significantly higher compared to pure salt crystallization. On the basis of analysis of Particle Size Distribution (PSD) and Critical Aggregation Concentration (CAC) of the polymer, it was proposed that the hydrophobic particles of macromolecules created from polymeric aggregates served as templates for the formation of hollow crystals. Their purity was verified using thermogravimetric analysis (TGA), 1H NMR, and XRD. Only trace amounts of polymer were found in the crystalline product.

## 1. Introduction

The substances within solutions play an important role during crystallization processes [1,2]. Depending on whether their presence was intentional or not, they are called admixtures or impurities, respectively. Since it is extremely easy to contaminate crystallizing samples, pure homogenous primary nucleation is rarely encountered [3]. As even the absorption of gases from the atmosphere may impact crystallization, one usually deals with heterogeneous primary crystallization [3,4,5]; whereas, in the case of industrial processes, the solutions are purposefully seeded, which promotes secondary nucleation, thus leading to crystalline product of desired size distribution [3]. At the present moment, the application of polymers as tool in the crystallization process of different compounds is becoming more and more popular [6]. One may generally distinguish three main fields of application. Firstly, the polymers may create a confined space within which the crystallization will occur [7,8,9]. For example, pores in microgels may act as microreactors which are characterized with very specific properties, not achievable in the conventional way. Secondly, one may encounter crystallization on the polymeric surfaces, or, in other words, the phenomenon of nucleation induction [10,11,12]. Here, different kinds of membranes or nanocomposites may be used for the stimulation of the nucleation process, creating hetero-nuclei sites. Finally, free macromolecules suspended in solution may act as nucleation inhibitors [13,14,15]. Moreover, they may screen specific faces of the growing crystals, thus influencing their polymorphism, habit, and even surface features. This research topic is quite vivid, and some spectacular results are achieved.

It is worth noting that multiple works are dedicated to the application of poly(acrylic acid) as an admixture in crystallization of various substances [16,17,18,19]. However, investigations of stimuli-responsive macromolecules in crystallization processes are uncommon [20]. In our previous work, we postulated a hypothesis that the phase transition of an Upper Critical Solution Temperature (UCST) macromolecule may destabilize a solution in the metastable state and, thus, initialize the nucleation process [21]. However, the experimental data showed that the hydrophilic/hydrophobic changes did not start the nucleation of crystals, and its onset had no impact on the nucleation temperature; however, this phenomenon impacted the growth of the crystals significantly. Therefore, we decided to explore this issue in more detail using star polymers.

Star-shaped polymers belong to the group of branched macromolecules, composed of a core located in the center of a macromolecule with chains/arms radiating from it. The physicochemical properties of star-shaped polymers depend on such parameters as: the number and length of arms as well as their structure and nature (hydrophilic and/or hydrophobic). Due to their branched structure, they have lower actual viscosities, hydrodynamic volumes, vitreous radii, and phase transition temperatures (melting point for crystalline polymers and glass transition temperatures for amorphous polymers) than their linear analogs with the same molecular weight [22]. The physicochemical properties of stars contributed to the interest in this type of compounds, e.g., as additives to lubricants [23], as additives to printing inks [24], and cosmetic dispersants [25], as well as for pharmaceutical use, where they could act as drug carriers [26]. However, it has to be emphasized that the application of star-shaped polymers in the controlled crystallization of salt was not investigated until now.

The issue of hollow crystal synthesis was already investigated in the previous century [27]. At the present moment, one may find more publications dedicated to this topic. For example, drugs such as fenoprofen calcium dehydrate or flufenamic acid have been crystallized as hollow crystals due to the lamellar structure formation [28,29]. These drugs were shown to have the advantage of improved dissolution and compressibility comparing to the plain ones. A surfactant-mediated co-reprecipitation method was used for creation of co-crystals comprised of 9-methylanthracene-1,2,4,5-tetracyanobenzene and naphthalene-1,2,4,5-tetracyanobenzene. According to the authors, such structures could be used for drug delivery systems or optoelectronics [30]. Wang et al. used single NaCl crystals as a water-soluble template for the formation of SiO_2_ and TiO_2_ hollow structures; however, they suggest other functional materials such as Al_2_O_3_, ZrO_2_, or SnO_2_ could be manufactured using the same approach [31]. Zn-nanorod-based hollow micro-hemispheres were obtained by the thermal thermolysis of a Zn(en)_2_^2+^ precursor in the presence of poly(sodium 4-styrenesulfonate). In this case, firstly, polymer-stabilized colloidal nanoclusters were generated, which formed secondary spherical particles. These, in turn, aggregate eventually, forming multipolar-shells at the cost of the small nanoclusters placed within the structure [32]. The Ostwald ripening phenomenon with the aid of polymers was used for the creation of CaTiO_3_ and Zn-hollow crystal [33,34].

**Figure 1:**
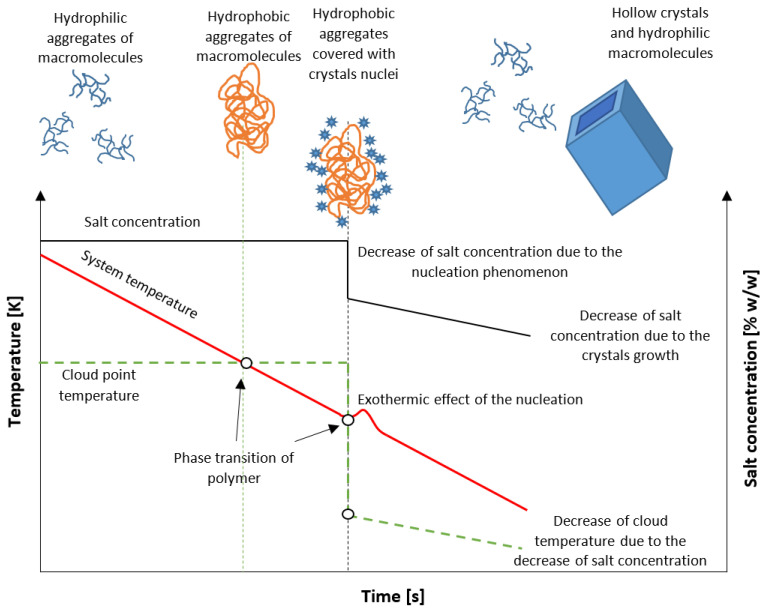
The postulated mechanism of crystallization.

In this work, we propose a new method of isohydric crystallization by solution cooling, allowing one to obtain hollow structure crystals. For this purpose, the unique properties of stimuli-responsive star amphiphilic polymers are used. The idea is presented in Figure 1. At the beginning of the process, the solution is at a stable state below the solubility curve. Additionally, an admixture in the form of UCST star polymer is present at a concentration sufficient to achieve critical aggregation concentration (CAC). The macromolecules form hydrophilic, freely floating assemblies which interact with ions present in the solution. In our previous work, it was proved that the application of poly(acrylic acid) at lower values of pH significantly broadens the Metastable Zone Width (MZW) [21]. Moreover, the Cloud Point Temperature (T_CP_) of polymer may be designed in such a way that the hydrophilic/hydrophobic transition will occur at any desired moment of the process. On the other hand, the location of T_CP_ had no impact on the occurrence of the nucleation process. These facts create favorable conditions for an unusual route of crystallization. If the phase transition takes place during the cooling of the solution, the macromolecules create insoluble templates around which nuclei will be gathered. Since extremely high supersaturation will be achieved, numerous nuclei will be bonded to the surface. Their subsequent growth will inevitably result in the aggregation of crystalline matter. Simultaneously, due to the drop of solute concentration, the macromolecules will change their state from hydrophobic to hydrophilic, once again creating a hollow structure.

## 2. Materials and Methods

### 2.1. Materials

*Tert*-butyl acrylate (*t*BuA, 99%, Alfa Aesar, Warsaw, Poland), methyl acrylate (MA, 99%, Sigma-Aldrich, Poznań, Poland), and anisole (Alfa Aesar, 99%) were dried over molecular sieves and stored under nitrogen. Copper(I) bromide (CuBr, 98%, Fluka, Steinheim, Germany) was purified by stirring in glacial acetic acid followed by filtration and washing with ethanol and diethyl ether, respectively. Next, the solid was dried under vacuum. Potassium chloride (KCl, VWR Chemicals, Gdansk, Poland) and *N*,*N*,*N*′,*N*″,*N*″-pentamethyldiethylenetriamine (PMDETA, 99%, Sigma-Aldrich, Poznan, Poland) were used as received. Pentaerythritoltetrakis(2-bromoisobutyrate) (PTLBr) was obtained according to a procedure described previously [35].

### 2.2. Synthesis of Polymer

Synthesis of 4-armed poly(acrylic acid-*co*-methyl acrylate) P(AA-*co*-MA) was realized by Atom Transfer Radical Polymerization (ATRP). CuBr (36.7 mg; 0.26 mmol), *t*BuA (15 mL; 102.4 mmol), MA (0.061 mL; 0.7 mmol), PMDETA (53 µL; 0.26 mmol), and anisole (4.5 mL; 30 vol% of monomers) were placed in a Schlenk tube (50 mL). After three freeze–pump–thaw cycles, pentaerythritoltetrakis(2-bromoisobutyrate) (PTLBr) (622.2 mg; 0.85 mmol/4 initiating sites) was added, and the Schlenk tube was sealed and placed in an oil bath thermostatted at 70 °C. After 22 h, the reaction was stopped by exposing the reaction mixture to air. Then, CH_2_Cl_2_ was added to the reaction mixture, which was subsequently passed through a neutral alumina column to remove the copper catalyst. After removing the solvents via a rotary evaporator, the remaining solution was poured into cold toluene. The precipitated copolymer was isolated by decantation and dried under vacuum at room temperature to a constant mass. Yield: 80%, M_n,calc_ = 15,700 g/mol, M_n,SEC_ = 16,200 g/mol, M_w_/M_n_ = 1.16. The next step was the acidolysis of the obtained copolymer using a procedure reported previously [36].

Conversions of monomers in (co)polymerizations were determined by gas chromatography. The chromatograph (6850 Network GC System, Agilent Technologies) was equipped with a flame ionization detector. Injector and detector temperatures were kept constant at 250 °C (conditions: depending on the solvent used for polymerization—anisole was used as an internal standard, column initial temperature 40 °C; column final temperature 200 °C). The conversions were calculated by detecting the decrease in the monomer peak area relative to the internal standard peak area. The SEC analysis was performed using size exclusion chromatograph (SEC, 1100 Agilent 1260 Infinity, Perlan Technologies, Warsaw, Poland) equipped with autosampler, degasser, isocratic pump, thermostatic box for columns, and differential refractometer MDS RI Detector (Perlan Technologies, Warsaw, Poland). Addon Rev. B.01.02 data analysis software (1st edition, Agilent Technologies) was used for data collection and processing. The SEC-calculated molecular weight was based on calibration using linear polystyrene standards (580–300,000 g/mol). A pre-column guard, 5 µm 50 mm × 7.5 mm, and PLGel, 5 µm MIXED-C 300 mm × 7.5 mm (Perlan Technologies, Warsaw, Poland), were used for separation. The measurements were carried out in tetrahydrofuran (THF) suitable for high performance liquid chromatography (HPLC grade) as the solvent at 40 °C with a flow rate of 0.8 mL/min. Further, 1H nuclear magnetic resonance (NMR) spectra were collected on Varian Inova 600 MHz spectrometer (Perlan Technologies, Warsaw, Poland) at 26 °C using tetramethylsilane (TMS) as an internal standard and 99.8% CDCl_3_ (in case of P(*t*BuA-*co*-MA) or 99.8% CD_3_OD (in case of P(AA-*co*-MA) as the solvents.

### 2.3. Measurement Procedures

Thermogravimetric analysis (TGA) of samples was conducted using a TA instruments TGA Q50. TGA was conducted between 30 and 700 °C at a heating rate of 10 °C∙min^−1^ in a 40 µL platinum pan under a nitrogen flow.

The CAC was determined using pyrene as a hydrophobic probe. The concentration of copolymers varied from 1 × 10^−3^ to 1 mg/mL. The pyrene solution (5 µM) in ethanol was added to a series of vials which were further stirred at room temperature in darkness for 24 h before measurement. The fluorescence emission spectra (λ_ex_ = 337 nm) of polymer/pyrene solutions were measured on Hitachi F-2500 fluorescence spectrophotometer. The CAC value was defined as the point of intersection of two lines in the plot of intensity ratio (I1/I3) from pyrene emission spectra versus the logarithm of the polymer concentration (logC, where C is a concentration in mg/mL).

The aggregation temperatures (T_agg_) of pure polymer aqueous solutions and solutions with 27% KCl were determined by dynamic light scattering (DLS) using a Malvern Zetasizer-S90. The aqueous solutions of polymers (0.25% *w/w*) or solutions of polymers (0.25% *w/w*) with KCl (27% *w/w*) were placed in quartz cuvettes which were then located in a thermostated chamber. The measurement temperature ranged from 27 to 45 °C with a heating rate of 2 °C/min.

The crystallization was realized in a standard quartz cuvette. The laboratory setup, comprising of a Cary 60 spectrophotometer by Agilent (MS Spectrum, Warsaw, Poland) and a TC-1 temperature controller by Quantum Northwest coupled with dedicated software (MS Spectrum, Warsaw, Poland), was used for transmittance measurements. The quartz cuvette (10 mm optical path), a Pasteur pipette, and 7 mm magnetic stirrer were preheated in a laboratory dryer at least 10 K above the saturation temperature before each analysis. The Peltier holder for the cuvette was also preheated. All samples were freshly prepared before measurements. Initially, 20 g of sample was prepared at a temperature 10 K higher than the saturation temperature. Then, a solution volume of 3.5 mL was transferred using a Pasteur pipette to the quartz cuvette which was instantaneously placed in the Peltier holder. The stabilization of the sample took 30 min. During that stage, as well as during the absorbance readings, the stirrer was working at 1000 [RPM]. As the result, intense mixing and, therefore, homogenization of crystal suspension in the last stage of experiments was achieved. During experiments, three parameters were directly measured, i.e., temperature of the sample (using a temperature sensor placed inside the cuvette), the temperature of the holder, and transmittance. For all measurements, the 1 K/min cooling rate was maintained. This was a compromise between the cooling rates used for investigations of stimuli-responsive polymers, cooling rates expected for the crystallization process, and the time needed to perform the measurements. In the research, each experimental point was investigated at least 5 times (i.e., at least 5 separate, identical samples were prepared). If the obtained results were not consistent, additional trials were performed.

After the crystallization, the crystals were collected for further analysis. Each sample was filtered using a funnel with sintered filter under low pressure. The crystals were washed with methanol (analytical grade) in order to remove the remaining solution and prevent further crystallization. Next, the crystals were dried in a laboratory dryer and analyzed using Phenom ProX scanning electron microscope (SEM) by Thermo Fisher Scientific equipped with EDS add-on. Samples, before SEM analysis, were coated with a 10 nm gold layer under vacuum using sputter coater Quorum Q150R ES. The crystals growth was observed using Nikon inverted microscope ECLIPSE Ti with Nikon DS-Ti1c. In this case, a fresh sample was prepared prior to the experiments. The crystallization occurred due to natural cooling of the sample under the microscope.

The purity of the obtained crystalline product was also verified by XRD, ^1^H NMR and TGA. XRD analysis was performed using powder X-ray diffractometer Seifert 3003TT with Cu X-ray tube (kλ_1_ = 1.540598 Å, kλ_2_ = 1.544426 Å, kβ = 1.39225 Å). The 2θ scan range varied from 5° to 80°, with step = 0.05°. Further, ^1^H NMR spectra were recorded at 25 °C with the aid of 600 MHz Varian spectrometer. Solutions of known concentration of polymer and salt were prepared in D_2_O phosphate buffer solution (pD = 7.13). Solution of 3-(trimethylsilyl)propionic-2,2,3,3-d4 acid sodium salt (TSP) in D_2_O placed in melt-point capillary was used as an external reference. Standard ^1^H NMR spectra were acquired with the relaxation delay of 10 s. TGA thermograms were conducted using a TA instruments TGA Q50 at temperature range between 30 and 700 °C and a heating rate of 10 °C∙min^−1^. Samples were weighted and placed in a 40 µL platinum pan under a nitrogen flow.

In all experiments, the polymer concentration was equal to 0.25% *w/w*. Three different KCl concentrations were chosen to be investigated, that is, 27% *w/w*, 28% *w/w*, and 29% *w/w*; as corresponding saturation temperatures were relatively low, the laboratory glassware was operated with bare hands. Simultaneously, the nucleation temperature was sufficiently high, and the cuvette did not have to be washed with dry air to prevent moisture condensation. Three characteristic points in the concentration vs. temperature coordinate system corresponding to the T_CP_ were chosen for each concentration of KCl: below saturation curve (stable zone), within the metastable zone, and above the metastable zone (labile zone, i.e., no phase transition of polymer). The points below the metastable zone correspond to pH = 2.20, within the metastable zone to pH = 2.25, and above the metastable zone to pH = 2.35. The pH was adjusted with ±0.01 accuracy. In conclusion, a grid of 9 points was created on which position was determined based on trial-and-error methods.

## 3. Results and Discussion

### 3.1. Crystallization Course

**Figure 2:**
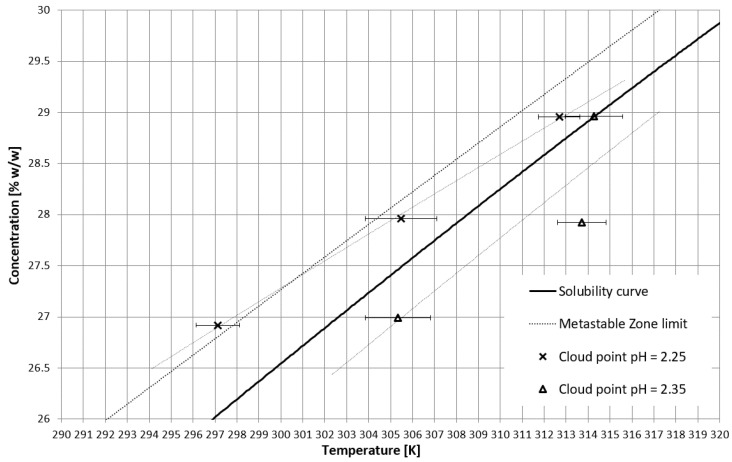
T_CP_ in the salt concentration vs. temperature coordinate system.

**Figure 3:**
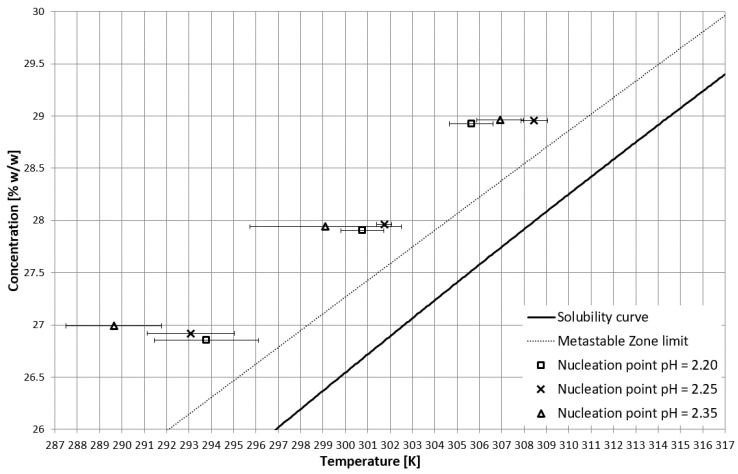
Nucleation points in the salt concentration vs. temperature coordinate system.

On the basis of trial-and-error methods, two pH values were determined in order to place the T_CP_ in two characteristic stages of crystallization, i.e., in the stable zone and in the metastable zone (Figure 2). They were equal to 2.35 and 2.25, respectively. The third value of pH, equal to 2.20, corresponded to the case when phase transition did not occur during the process (Figure 2 and Figure 3). In Figure 2, the dot line depicts the metastable zone limit achieved for pure KCl crystallization. Two additional lines represent the trend of change of the T_CP_ value, which is consistent with our previous research [21,22,23,24,25,26,27,28,29,30,31,32,33,34,35,36,37]. Figure 3 presents the nucleation points for each case. As can be easily noticed, the width of the metastable zone extended significantly compared to the standard process. Such a result could be expected, as poly(Acrcylic Acid) PAA was reported to inhibit the nucleation process and, thus, is used as an antiscalant agent [19,38,39], which was also proved in our previous study [21]. As in the case when linear PAA was used [21] in the discussed research, the location of the T_CP_ (the occurrence of the phase transition phenomenon) had no clear impact on the nucleation temperature. The nucleation occurred for very high supersaturations which resulted in high randomness of the process. Interestingly, the highest supersaturations were usually achieved when the macromolecules changed their properties from hydrophilic to hydrophobic in the stable zone; therefore, at the early stage of the process.

This fact ultimately disproves our initial hypothesis that phase transition and the presence of hydrophobic particles may destabilize the system in the metastable zone. Not only the fact that macromolecules transition did not trigger nucleation, but the continuous presence of hydrophobic particles also extended the width of the metastable zone. In our opinion, the electrostatic interactions between polymeric chains and ions present in the suspension surpass any other phenomena which took place during the process.

**Figure 4:**
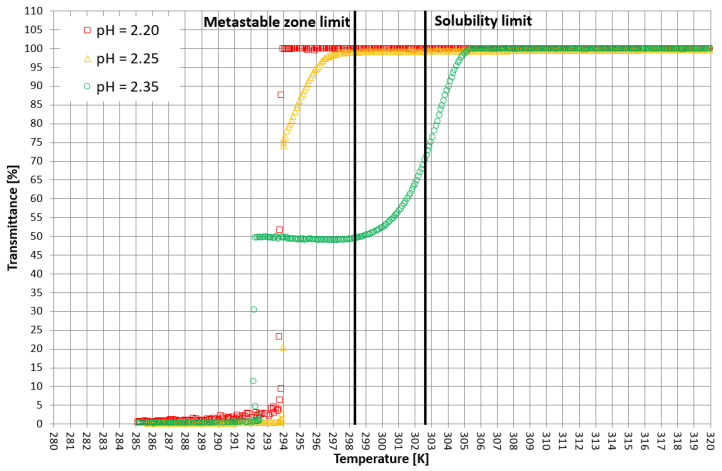
Exemplary crystallization courses for 27% *w/w* salt concentration in the transmittance vs. temperature coordinate system.

**Figure 5:**
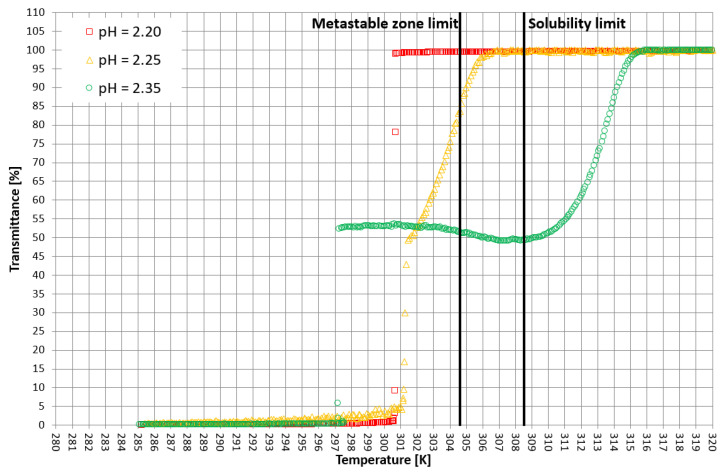
Exemplary crystallization courses for 28% *w/w* salt concentration in the transmittance vs. temperature coordinate system.

**Figure 6:**
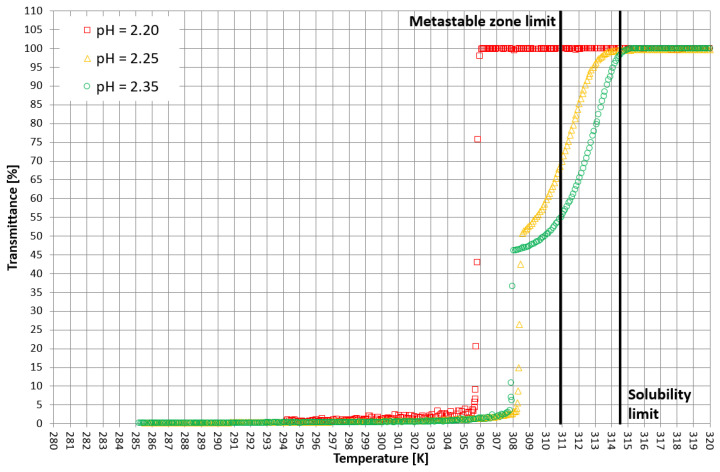
Exemplary crystallization courses for 29% *w/w* salt concentration in the transmittance vs. temperature coordinate system.

More detailed insight into this process comes from the analysis of the course of crystallization (Figure 4, Figure 5 and Figure 6). As it was stated above, for three KCl concentrations, three characteristic cases were considered: phase transition within the stable zone, phase transition within the metastable zone, and no phase transition. In Figure 4, Figure 5 and Figure 6, the “gentle” change of transmittance corresponds to the occurrence of hydrophobic particles of macromolecules. Simply speaking, the solution becomes cloudy. Then, the sudden drop of transmittance accompanied by temperature distortion represents the nucleation of KCl crystals. For the lowest considered value of pH, the phase transition did not occur; therefore, one may notice an instantaneous drop of transmittance in the sample. Surprisingly, when the T_CP_ was located in the stable zone, and thus the hydrophobic particles were mixed for a longer period of time, the increase in transmittance could be noticed. This fact may be related to the aggregation of macromolecules due to the hydrophobic interaction. This phenomenon was not noticed by us in the case of linear PAA [21].

### 3.2. Analysis Using Scanning Electron Microscopy

**Figure 7:**
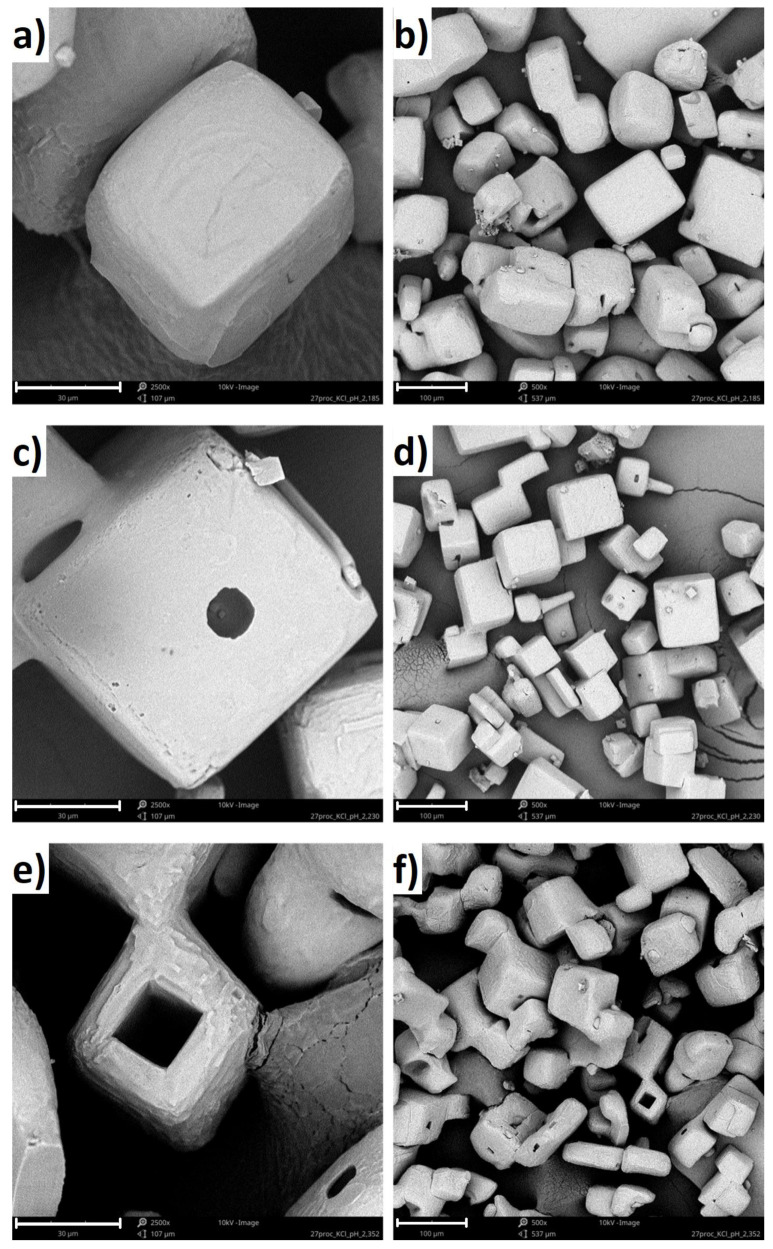
SEM images of KCl crystals obtained from 27% *w/w* solution for: (**a**) four-arm P(AA-*co*-MA) (from the solution at pH = 2.20, 30 μm scale bar), (**b**) four-arm P(AA-*co*-MA) (from the solution at pH = 2.20, 100 μm scale bar), (**c**) four-arm P(AA-*co*-MA) (from the solution at pH = 2.25, 30 μm scale bar), (**d**) four-arm P(AA-*co*-MA) (from the solution at pH = 2.25, 100 μm scale bar), (**e**) four-arm P(AA-*co*-MA) (from the solution at pH = 2.35, 30 μm scale bar), and (**f**) four-arm P(AA-*co*-MA) (from the solution at pH = 2.35, 100 μm scale bar).

SEM micrographs of KCl crystals recovered from the solutions after crystallization with polymers are shown in Figure 7. Based on the images, it can be concluded that the presence of the polymer in the solution significantly influenced the crystals growth mechanism. In the case of pure KCl solution, hopper-cubic crystals were obtained with relatively smooth faces and rounded edges [21]. The cavities at the center of the walls may be attributed to mother liquor occlusion, whereas the edge roundness results from mechanical attrition. Further, if the linear PAA was present in the solution, the crystal surface morphology changed noticeably [21]. The crystal had a cubic shape, with clearly defined edges. Finally, if the four-arm P(AA-*co*-MA) was used during crystallization, the hollow crystals emerged. In the literature, is has already been shown that NaCl and KCl can form hollow crystals (hopper-like crystals) in certain conditions [40]. In our opinion, in our case, coil to globule transition forced the aggregation of hydrophobic forms of macromolecules. Presumably, the polymeric aggregates served as templates for the KCl crystals growth. At this stage, our results suggested that, when the salt concentration decreased within the solution, the polymeric chains once again became hydrophilic, leaving empty space in the center of the crystal.

### 3.3. Critical Aggregation Concentration and Particle Size Distribution of Polymers

**Figure 8:**
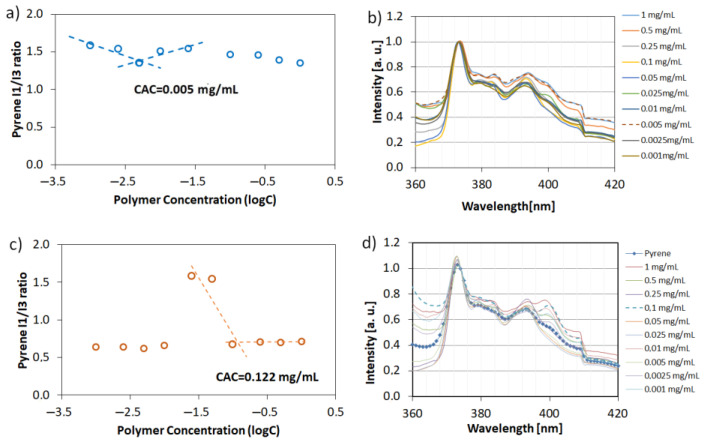
Plots of the intensity ratio I1/I3 from pyrene emission spectra versus the logarithm of polymer concentration with corresponding normalized fluorescence spectra: four-arm P(AA-*co*-MA) (**a**,**b**) and linear PAA (**c**,**d**).

In order to prove our hypothesis related to the mechanism of hollow crystals formation, the critical aggregation concentration (CAC) of PAA and P(AA-*co*-MA) were determined via an indirect method based on the measurement of pyrene fluorescence spectra in polymeric water solutions (Figure 8). In both series of experiments using linear PAA and four-arm AA/MA copolymer, the concentration of macromolecules was equal to C_pol_ = 3 mg/mL (which corresponds to 0.25% *w/w* concentration). Linear PAA, used in the previous studies [21], led to the formation of cubic crystals with well-defined sharp edges and a rough surface. However, no cavities in the walls of the crystals were noticed. The pyrene fluorescence spectra analysis proved that its concentration used in experiments was way above its CAC. What is more important is that the CAC value for four-arm AA/MA copolymer was approximately 24 times lower than in the case of linear PAA. It can be explained by the fact that branched copolymers have lower CAC values in comparison to their linear analogue and possess methyl acrylate units which are less hydrophilic than acrylic acid repeating units. Therefore, even in the hydrophilic form, four-arm AA/MA copolymer had a higher chance to create agglomerates of macromolecules. They, in turn, could aggregate further after phase transition due to the hydrophobic interactions.

**Figure 9:**
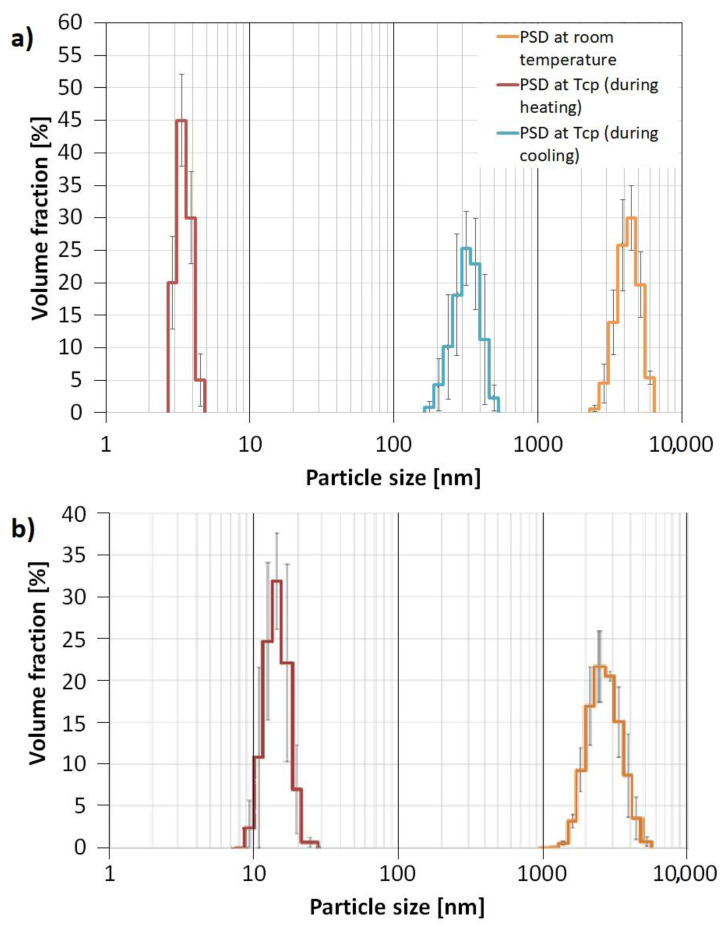
Particle Size Distribution for four-arm P(AA-*co*-MA) (**a**) and linear PAA (**b**).

The aggregates formation was proven by DLS analysis which showed that four-arm AA/MA copolymer solution with KCl created aggregates at 298.15 K with D_h_ = 4.145 µm, whereas particles of linear PAA had D_h_ = 2.900 µm (Figure 9). Since the concentration of both polymers in KCl solutions exceeded their CAC values, the formation of hollow KCl crystals during crystallization should have occurred in both cases. However, the experiments on the behavior of polymeric solutions with KCl performed by measuring their particle sizes in the function of temperature revealed that the four-arm polymer creates aggregates with higher Dh values at its T_CP_ during cooling (295 nm) in comparison to the heating mode (3.6 nm). In the case of linear PAA, regardless of whether the sample was heated or cooled, the aggregates at T_CP_ had sizes of approximately 15 nm. In our opinion, such hysteresis in size distribution resulted from the sedimentation of the biggest aggregates at the bottom of the cuvette. When the sample was heated once again, only the smallest particles were present in the bulk of the solution due to Brownian movement.

In the literature, one may find some works reporting the formation of hollow crystals. Mainly two different mechanisms of such a phenomenon are proposed. The first one is based on the Ostwald ripening. Yang et al. [33] proposed the mechanism in which poly(ethylene glycol) influenced the aggregation of CaTiO_2_ nanocrystals leading to the formation of spherical particles. Then, Ostwald ripening resulted in the formation of hollow structures. A similar approach to the formation of dumbbell-like ZnO hollow crystals was proposed by Yue at al. [34]. Here, poly(sodium 4-styrenesulfonate) was used as a crystal growth control agent. As in the previous case, the authors suggest that macromolecules influenced the aggregation process of colloidal particles, leading to the formation of larger structures. Dummbell-like twinning crystals were spontaneously created which underwent subsequent growth. Since the growth along the polar axis orientation was hampered, sheet-like ZnO was formed. The authors stated that further growth of crystals was based on the Ostwald ripening phenomenon. The second mechanism which may be found in the literature is based on the hampering of crystals wall-growth in a given direction by the presence of specific additives. For example, Zhang et al. [40] presented the formation of hopper-like crystals of sodium chloride. In this case, cyclohexane was used as the agent inhibiting the growth of one of the planes of crystals. In such a way, the cubic hopper-like crystals were created. However, it is worth emphasizing that, in order to achieve these unusual results, researchers had to use a complex reaction system.

In order to gain deeper insight into the growth process of KCl crystals, the phenomenon was recorded by us using optical microscope (Supplementary materials: Videos S1 and S2). Two characteristic features of the crystal growth may be noticed from the recordings. Firstly, the dendritic growth of crystal may be noticed (Video S1). This is the evident result of extremely high supersaturations achieved when the polymer was used (Figure 3) [3]. Secondly, the diffusion of microparticles and formation of larger crystals is clearly visible (Video S2). Thus, in our opinion, the phenomenon investigated in this paper is more similar the first of the abovementioned mechanisms of hollow crystals formation. We think that the crystal growth mechanism occurs according to birth and spread model [3]. The growth of the next layer is initialized at the edges of crystals. Due to the thermodynamic barrier generated by the presence of macromolecules, a hollow space is created at the center of the crystal. Such behavior was not noticed when the linear PAA was used as an additive [21]. In that case, crystals had a rough surface which proves that the aggregation process played an important role in their formation. The absence of cavities is the result of the size of macromolecules present in the solution (or rather their aggregates).

### 3.4. Purity of Crystalline Product

**Figure 10:**
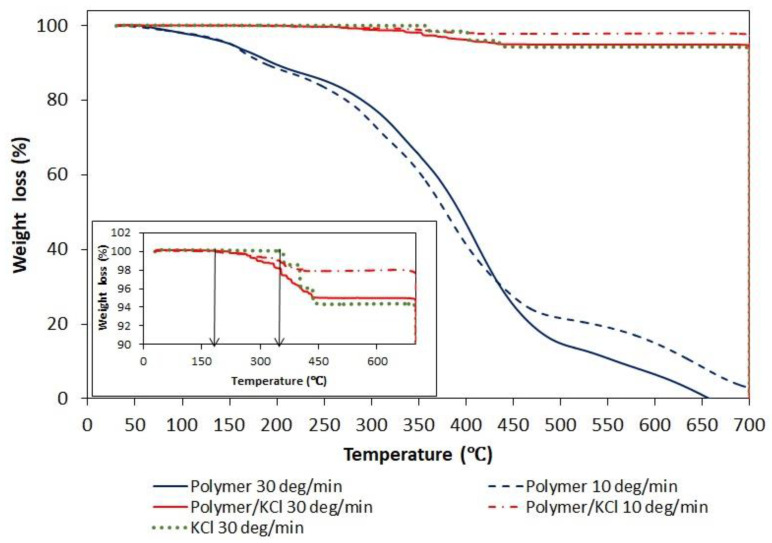
Thermogravimetric analysis traces under nitrogen of pure P(AA-*co*-MA), pure KCl, and KCl crystals recovered after crystallization with P(AA-*co*-MA).

Since, in order to create hollow crystals, the phase transition of polymer had to occur above the solubility temperature of KCl, the polymer content in the crystalline product was determined. The presence of residual polymeric particles occluded in KCl crystals was suspected after TGA analysis (Figure 10). The thermogram of a pure AA/MA copolymer shows three decomposition stages similar to ones described in the literature for PAA. The first decomposition stage (40–180 °C) is attributed to the loss of absorbed water molecules. The second decomposition stage (180–250 °C) corresponds to the dehydratation and decarboxylation of the polymer. The third decomposition stage (300–500 °C) is a result of the final degradation of the polymer. As shown in the inserted figure representing enlarged region of TGA curves in the range 90–100% of weight loss, the tenuous differences appear in the decomposition curve of pure KCl and KCl recovered after crystallization in the presence of a polymer. The temperature at which the decomposition of KCl recovered after crystallization starts is shifted toward lower temperatures and overlaps with the end of the first decomposition stage curve of pure polymer. In order to confirm or exclude the presence of polymer in the KCl crystals, XRD and NMR analyzes were performed.

**Figure 11:**
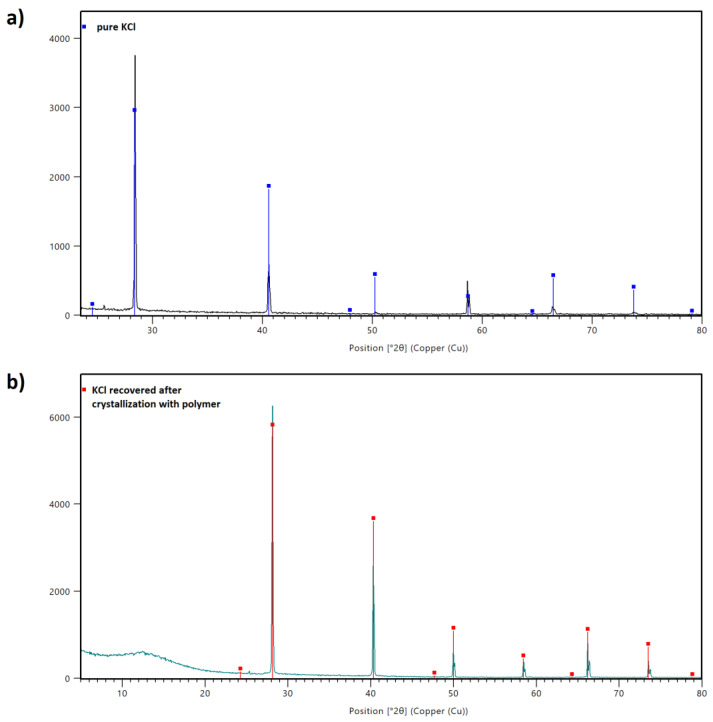
XRD patterns of sample prepared from pure KCl (**a**) and KCl recovered after the crystallization process with P(AA-*co*-MA) (**b**).

The XRD results are given in Figure 11. The first pattern shows the distinct peaks corresponding solely to the crystalline phase identified as KCl (Figure 11a). The analysis performed for the KCl sample recovered after crystallization in the presence of AA/MA copolymer shows the amorphous phase between 5° and 20° of 2θ coming from the polymer. The diffraction peaks for KCl were registered between 29° and 80° of 2θ.

**Figure 12:**
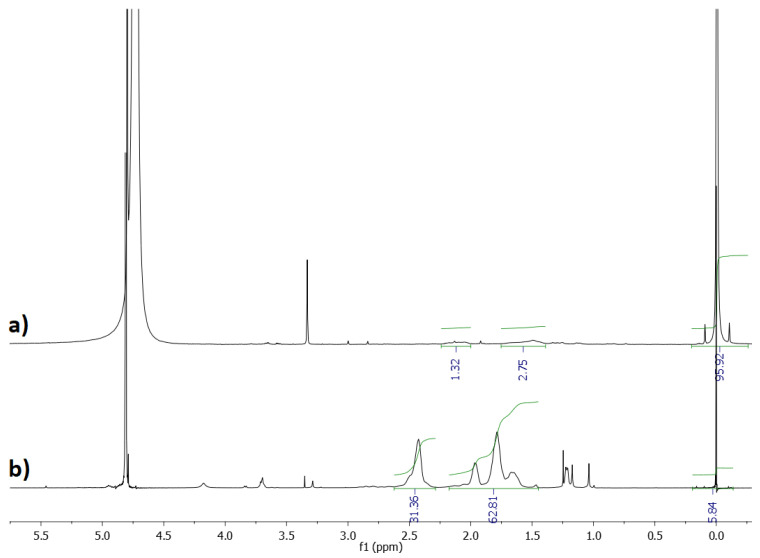
^1^H NMR (600 MHz, D_2_O) spectra of KCl recovered after the crystallization process (**a**) and P(AA-*co*-MA) (**b**).

Figure 12 shows the ^1^H NMR spectra of KCl recovered after the crystallization process and pure P(AA-*co*-MA) copolymer sample. On that basis, it was calculated that the KCl crystals contained about 0.3% *w/w* of the polymer. The result was satisfying, especially considering that the amount of the polymer which was used in the initial solution was 0.93% by weight of KCl.

## 4. Conclusions

The experimental research proved the postulated hypothesis—stimuli responsive polymers may be used as templates in the crystallization process, leading to the formation of hollow crystals. It must be emphasized that considering the T_CP_ and molecular weight of polymers is not sufficient in order to predict the outcome of the crystallization process. Equally important is the behavior of macromolecules in the solution. The four-arm AA/MA copolymer used in this research had a significantly lower molecular weight compared to PAA, which was used previously [21]. Yet, its specific structure resulted in a CAC value at a considerably lower level. As a result, using exactly the same process conditions (excluding pH value which had to be set individually) in the case of linear PAA, crystals with sharp edges and rough surfaces were created, whereas, in the case of four-arm AA/MA, copolymer hollow crystals with smooth surfaces were obtained. Still, some common features of the application of stimuli responsive polymers may be distinguished. First of all, the broadening of the metastable zone width. The nucleation occurred at temperatures noticeably lower compared to the crystallization of KCl from pure solution. Secondly, the phase transition of polymers did not influence the nucleation temperature. No matter at which stage the change from hydrophilic to hydrophobic state occurred, the nucleation always took place beyond the metastable limit of the conventional process. The next step of the presented research will be the scale-up process and optimization of process conditions considering the purity of the final product.

## Data Availability

The data presented in this study are available on request from the corresponding author.

## Supplementary Materials

The following supporting information can be downloaded at: https://www.mdpi.com/article/10.3390/ma15228240/s1, Video S1: Dendritic growth of crystals; Video S2: Formation of larger crystals.
